# Gate-Tunable Band Edge in Few-Layer MoS_2_

**DOI:** 10.1021/acs.nanolett.5c01998

**Published:** 2025-06-22

**Authors:** Michele Masseroni, Isaac Soltero, James G. McHugh, Igor Rozhansky, Xue Li, Alexander Schmidhuber, Markus Niese, Takashi Taniguchi, Kenji Watanabe, Vladimir I. Fal’ko, Thomas Ihn, Klaus Ensslin

**Affiliations:** † Solid State Physics Laboratory, ETH Zürich, 8093 Zürich, Switzerland; ‡ Department of Physics and Astronomy, 5292 University of Manchester, Oxford Road, Manchester M13 9PL, United Kingdom; ¶ National Graphene Institute, 5292 University of Manchester, Booth Street East, Manchester M13 9PL, United Kingdom; § Research Center for Materials Nanoarchitectonics, 52747 National Institute for Materials Science, 1-1 Namiki, Tsukuba 305-0044, Japan; ∥ Research Center for Electronic and Optical Materials, 52747 National Institute for Materials Science, 1-1 Namiki, Tsukuba 305-0044, Japan; ⊥ Quantum Center, ETH Zürich, 8093 Zürich, Switzerland

**Keywords:** MoS_2_, Shubnikov−de Haas oscillations, field effect, band edge alignment, layer polarization, interlayer screening

## Abstract

Transition metal dichalcogenides (TMDs) have garnered significant research interest due to the variation in band edge locations within the hexagonal Brillouin zone between single-layer and bulk configurations. In monolayers, the conduction band minima are centered at the *K* points, whereas in multilayers, they shift to the *Q* points, midway between the Γ and *K* points. In this study, we conduct magnetotransport experiments to measure the occupation in the *Q* and *K* valleys in four-layer molybdenum disulfide (MoS_2_). We demonstrate electrostatic tunability of the conduction band edge by combining our experimental results with a hybrid *k*·*p* tight-binding model that accounts for interlayer screening effects in a self-consistent manner. Furthermore, we extend our model to bilayer and trilayer MoS_2_, reconciling prior experimental results and quantifying the tunable range of band edges in atomically thin TMDs.

## Graphical abstract

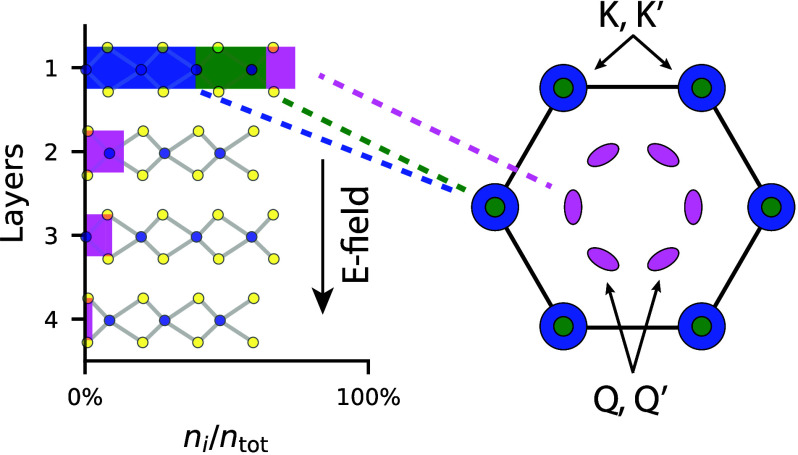

Transition metal dichalcogenides (TMDs) display unique electronic^1–3^ and optical properties.^4,5^ A key feature of TMDs is the presence of multiple valleys in their band structure, with the conduction band hosting valleys at both the *K* points and the *Q* points (located midway between the Γ and *K* points) of the first Brillouin zone, while the valence band exhibits valleys at the *K* points and the Γ point.^6^ The relative energy levels of these valleys are strongly influenced by the number of layers, leading to layer-dependent modifications in the band structure.^7,8^

In our previous studies, we systematically investigated the conduction band of monolayer,^9^ bilayer,^10^ and trilayer MoS_2_.^11^ We found that electron transport predominantly occurred via the *K* valleys of the conduction band, while the occupation of *Q* valleys, predicted by theory,^12–17^ was not detected in our measurements. In these studies, we employed electrostatic gating to overcome the Schottky barrier that typically forms at the metal–MoS_2_ interface. However, the influence of the gate-induced electric field on the band structure, particularly the relative energy shift of different valleys,^18^ was not considered.

Here, we investigate four-layer MoS_2_, where we observe a gate-dependent band edge transition in the conduction band minima, shifting from the *Q* valleys at low gate voltages to the *K* valleys at higher voltages. This transition reveals the sensitivity of the conduction band to external electric fields, which we attribute to the differing atomic orbital compositions of the *Q* and *K* valleys as well as interlayer screening effects. Our findings not only demonstrate the coexistence of both *Q* and *K* valleys in biased four-layer MoS_2_ but also show how electrostatic gating can effectively tune the band structure in TMD-based devices. These results offer deeper insights into the electronic properties of multilayer MoS_2_ and present potential avenues for valley-selective device applications.

In this study, we use dual-gated, multiterminal devices, as shown in Figure 1a. The devices are heterostructures consisting of four-layer MoS_2_ encapsulated in hexagonal boron nitride (hBN), all obtained by mechanical exfoliation (fabrication details in Supplementary Note 1). We conducted the experiments on two samples: sample A, featuring a graphite bottom gate and a metallic top gate, and sample B, with metallic gates on both sides. The results presented in the main text are from sample A, which exhibits higher electron mobility and more pronounced Shubnikov—de Haas oscillations (SdHO). Similar results obtained for sample B are provided in the Supporting Information.

**Figure 1:**
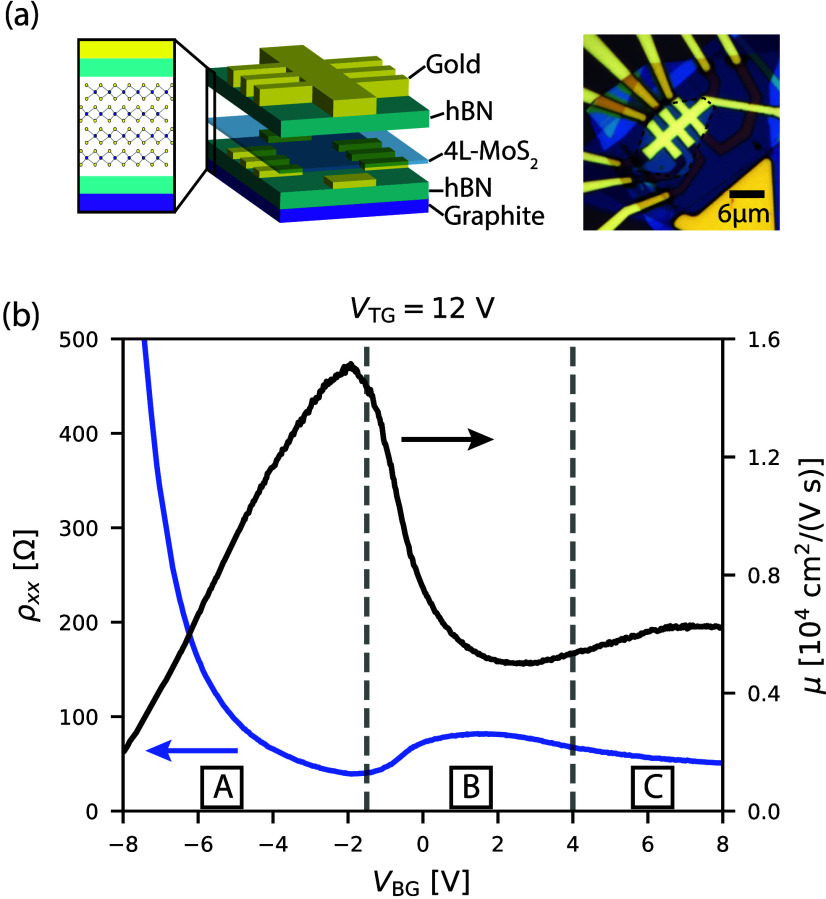
(a) Schematic view (left) and optical image of the sample (right). In the right panel, the MoS_2_ layer is outlined with a black dashed line. The gate defines a conducting channel of width *W* = 2 μm. The distance between adjacent contacts is *L*_C–C_ = 3 μm. (b) Four-terminal resistivity (blue) and mobility (black) as a function of the bottom gate voltage at *V*_TG_ = 12 V. The vertical dashed lines mark the voltage at which additional bands are populated. This measurement was performed at a temperature of *T* ≈ 100 mK.

One of the challenges of electronic transport experiments in MoS_2_ devices is achieving ohmic contacts. This issue is effectively addressed by employing a sample geometry with gated metallic contacts.^2,9,19–21^ In such samples, finite conductance at cryogenic temperatures is achieved only at relatively large top gate voltages (*V*_TG_), which are required to overcome the Schottky barrier at the metal–MoS_2_ interface (see also Supplementary Note 2).

For this reason, in our experiments, we fix the top gate voltage and tune the density with the bottom gate voltage (*V*_BG_), which does not affect the electron density in the region of the contacts due to the screening provided by the metallic contact pads. This enables us to maintain low-resistance (∼1 kΩ) ohmic contacts for all densities in the Hall bar. However, this sample geometry restricts the parameter space spanned by the gates. As a consequence, the samples are typically operated under a finite electric displacement field (*D*), which affects the electron distribution across layers in multilayer MoS_2_, potentially affecting their electronic properties.

In Figure 1b, we present the resistivity (blue) as a function of *V*_BG_ measured at *V*_TG_ = 12 V and *T* ≈ 100 mK. The resistivity reveals a nonmonotonic dependence on *V*_BG_, which is also evident in the transport mobility (black). The mobility increases roughly linearly in the voltage range *V*_BG_ < −2 V (regime A in the figure), reaching a peak value of μ_peak_ ≈ 1.5 × 10^4^ cm^2^ V^–1^ s^–1^ at an electron density of approximately 1 × 10^13^ cm^–2^. Further increasing *V*_BG_ results in a significant decrease in mobility, occurring close to the boundary between regimes A and B. We attribute this decrease to the occupation of additional bands in regime B, as determined by the density analysis presented below. We note that the boundaries between the various regimes do not necessarily coincide with the onset of mobility reduction, which begins already within regime A. This discrepancy arises because the mobility is influenced not only by band population but also by additional scattering mechanisms—such as those induced by defect states—that may become active even before mobile states in the second band are occupied.

To investigate the contribution of the different bands in four-layer MoS_2_, we examine the data obtained at finite magnetic fields. In Figure 2a, we present the magnetoresistance Δρ_*xx*_/ρ_0_ = [ρ_*xx*_(*B*) – ρ_*xx*_(0)]/ρ_*xx*_(0) as a function of *V*_BG_ measured at *V*_TG_ = 12 V. This measurement reveals an intricate pattern that originates from overlapping Landau fans. The observation of multiple Landau fans confirms the existence of multiple electronic bands.

**Figure 2:**
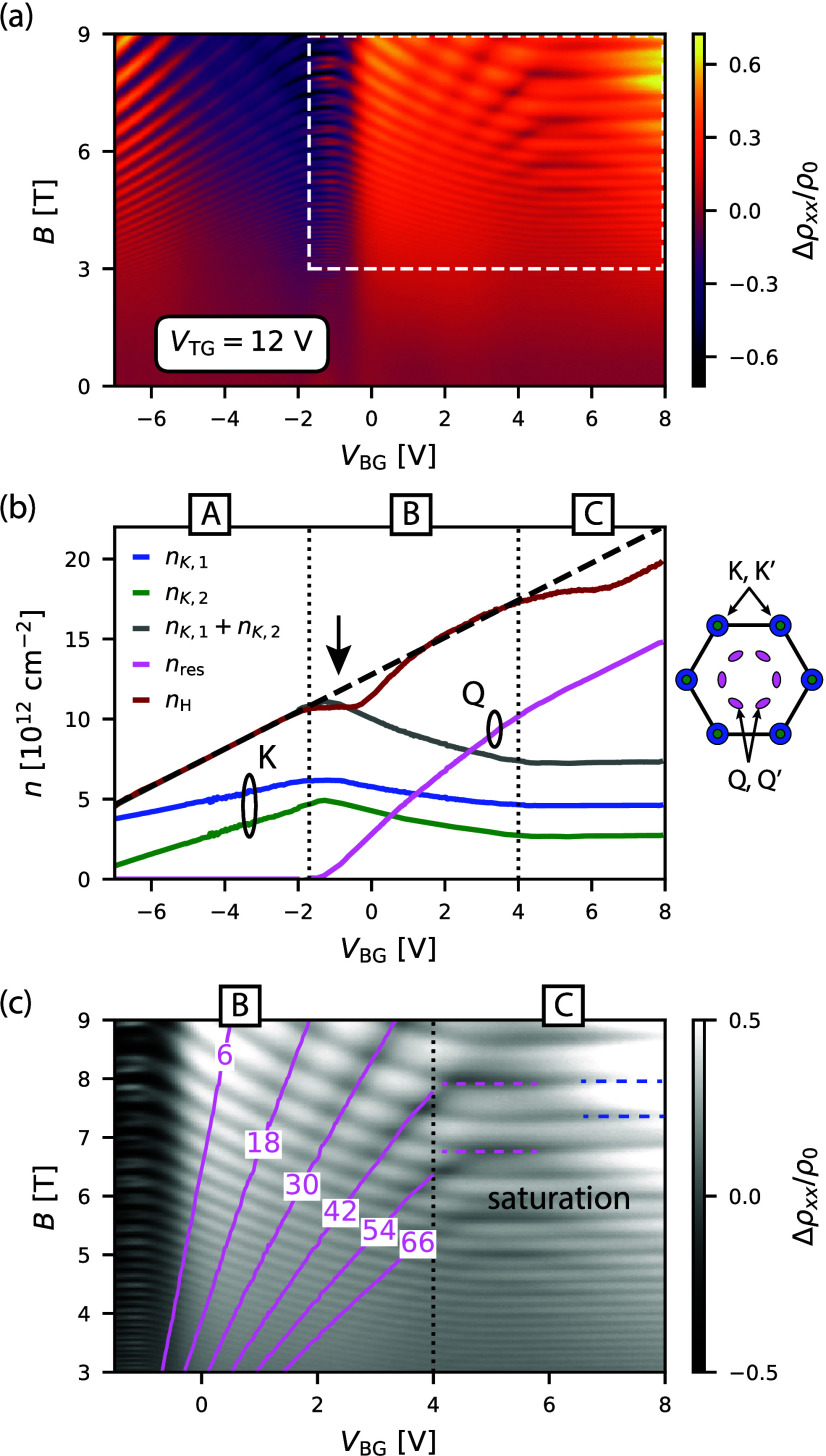
(a) Δρ_*xx*_/ρ_0_ = [ρ_*xx*_(*B*) – ρ_*xx*_(0)]/ρ_*xx*_(0) plotted as a function of *V*_BG_ and *B* at *V*_TG_ = 12 V and *T* ≈ 100 mK. The white dashed frame highlight the magnified image displayed in panel c. (b) Electron densities versus *V*_BG_: total density *n*_tot_ (black dashed), Hall density *n*_H_ (red), SdHO-derived *K* valley densities *n*_*K*,1_ and *n*_*K*,2_ (blue and green, respectively), and residual density *n*_res_ = *n*_tot_ – *n*_*K*,1_ – *n*_*K*,2_ (pink). Note that *n*_H_ is extracted from the high-field linear regime of *R*_*xy*_(*B* > 1 T), where multiband effects are suppressed. See Supplementary Note 4 for details on the Hall effect analysis. The inset shows the Brillouin zone of MoS_2_ with colored pockets indicating valley occupation. (c) Close-up of a portion of panel a, showing the Landau fan (pink lines) for *n*_res_ with a degeneracy *g* = 12. Horizontal dashed lines in regime C indicate valley density saturation.

Each of those bands has a corresponding electron density that determines the frequency of the SdHO. We extract the densities by computing the fast Fourier transform (FFT) of Δρ_*xx*_(*B* ^–1^) at each value of *V*_BG_ (details in Supplementary Note 3) and plot the resulting densities in Figure 2b. In the voltage range *V*_BG_ < −1.7 V (regime A), the Fourier analysis reveals two electron densities, *n*_*K*,1_ and *n*_*K*,2_ (blue and green curves, respectively), both with a similar gate voltage dependence. The densities are calculated using the relationship *n*_*i*_ = *g*_*i*_ *ef*_*i*_/*h*, where *f*_*i*_ is the frequency, *g*_*i*_ is the degeneracy of band *i*, *e* is the elementary charge, and *h* is Plank’s constant. By comparing the sum *n*_*K*,1_ + *n*_*K*,2_ (gray curve) to total density *n*_H_ obtained from the Hall effect measurements (red curve), we determine a degeneracy of *g* = 2 for both bands. This degeneracy suggests that the bands are centered at the *K* points of the Brillouin zone.

The density difference, *n*_*K*,1_ – *n*_*K*,2_, arises from the spin–orbit splitting of the *K* valley. By linear extrapolation, we estimate *n*_*K*,1_ ≈ 3.2 × 10^12^ cm^–2^ at the onset of density *n*_*K*,2_, which aligns with the density required to populate the upper spin–orbit split band at the *K* points observed in previous studies in monolayer,^9^ bilayer,^10^ and trilayer^11^ MoS_2_. This further supports our interpretation.

Now, we turn our attention to regime B in Figure 2b, where an additional band begins to be populated. This is evident in three distinct aspects. The first is the emergence of another Landau fan in Figure 2a (highlighted by the white dashed frame), with its origin around *V*_BG_ ≈ −1.7 V. At the same gate voltage, the Hall density exhibits a plateau (marked by an arrow), which we attribute to the filling of defect states at the bottom of the newly occupied band. This results in the localization of electrons at the defects, preventing them from contributing to the Hall effect (see also Supplementary Note 4 for further discussion). Third, the sum *n*_*K*,1_ + *n*_*K*,2_ is no longer equal to the Hall density, implying the presence of an extra electron pocket.

We determine the degeneracy of the newly occupied band by calculating the residual density using the relation *n*_res_ = *n*_tot_ – *n*_*K*,1_ – *n*_*K*,2_ (depicted by the pink curve in Figure 2b) and using it to predict the Landau fan according to the equation

1 $$B_{m}\left(V_{BG}\right)=\frac{hn_{res}\left(V_{BG}\right)}{egm}$$

where *m* is an integer representing the Landau level index and *g* is the band degeneracy. In Figure 2c, we plot the calculated Landau fan where we match the minima of Δρ_*xx*_/ρ_0_ by assuming *g* = 12. This large degeneracy suggests that the residual electron density originates from the *Q* valley, which has a 6-fold valley degeneracy. To achieve a total degeneracy of 12, the bands must be spin degenerate, implying inversion symmetry. However, while inversion symmetry is preserved in unbiased MoS_2_ samples with an even number of layers, the large displacement field in regime B (*D* ∼ 1 V/nm) is expected to break this symmetry.

To gain further insight, we implement a model to analyze the layer-resolved valley densities in four-layer MoS_2_ under various displacement fields and total electron densities. This model incorporates self-consistent screening effects produced by charge accumulation in each layer, allowing us to capture the impact of interlayer screening on the electron distribution. We construct hybrid *k*·*p* tight-binding Hamiltonians to simulate the electronic structure of the *K* and *Q* valleys. These Hamiltonians were initially parametrized using density functional theory (DFT) calculations (see Supplementary Notes 8 and 9) and included an on-site term to account for electrostatic band bending due to external fields. Notably, DFT results confirmed the absence of hybridization between the *K* valleys of different layers, consistent with prior results.^10^ According to the findings in ref 22, the spin–orbit splitting between *K* bands, Δ_SO_^*K*^, and the monolayer energy offset between *K* and *Q* valleys, *E*_*KQ*_, undergo significant renormalization due to electron–electron exchange interactions. In line with this framework, we considered both of these quantities to be dependent on layer density (see Supplementary Note 10).

The predictive accuracy of the model is based on two essential parameters: *t*_0_, the interlayer tunnel coupling for *Q* valleys, and *E*_*KQ*_. However, DFT-derived parameters exhibit a pronounced sensitivity to subtle variations in the MoS_2_ interlayer spacing and lattice constants (see Figure 11 in Supplementary Note 9). This sensitivity introduces considerable uncertainty into their precise values, and in fact, theoretical values alone did not align with our experimental results. To overcome this limitation, we varied *t*_0_ and *E*_*KQ*_ semiempirically to achieve a better fit to the observed layer and valley densities. Remarkably, we found that the distribution of electron densities across layers and valleys is highly sensitive to variations in *t*_0_ and *E*_*KQ*_ (see Supplementary Note 11). This characteristic suggests a novel experimental approach for determining interlayer tunnel coupling, which may also be extended to other TMDs. The optimized parameters for four-layer MoS_2_ determined through this process are *t*_0_ = 0.16 eV and *E*_*KQ*_ = 0.21 eV. These values are physically reasonable, lie within the range suggested by DFT, and provide significantly better agreement with the experimental gate-dependent valley and layer density evolution.

The band densities obtained from our model are shown in Figure 3a as a function of *V*_BG_ for *V*_TG_ = 12 V, allowing a direct comparison with the experimental densities in Figure 2b. The model identifies three distinct operating regimes controlled by the bottom gate voltage, reflecting the experimental observations. In regime A, where the voltage is applied asymmetrically between the top and bottom gates, the density is concentrated entirely in the *K* valleys of the top layer (see the layer polarization shown in Figure 3b). In regime B, where the bottom gate voltage is close to zero, the *Q* valleys begin to fill with electrons, and charge is transferred from the *K* valley to the newly occupied band. Since the *Q* valley states are hybridized between layers, the density is distributed across layers, favoring layers with a lower potential energy. In regime C, the *K* valleys in the bottom layers are filled, leading to saturation of the *K* (top layer) and *Q* valley densities, as observed in the experiment.

**Figure 3:**
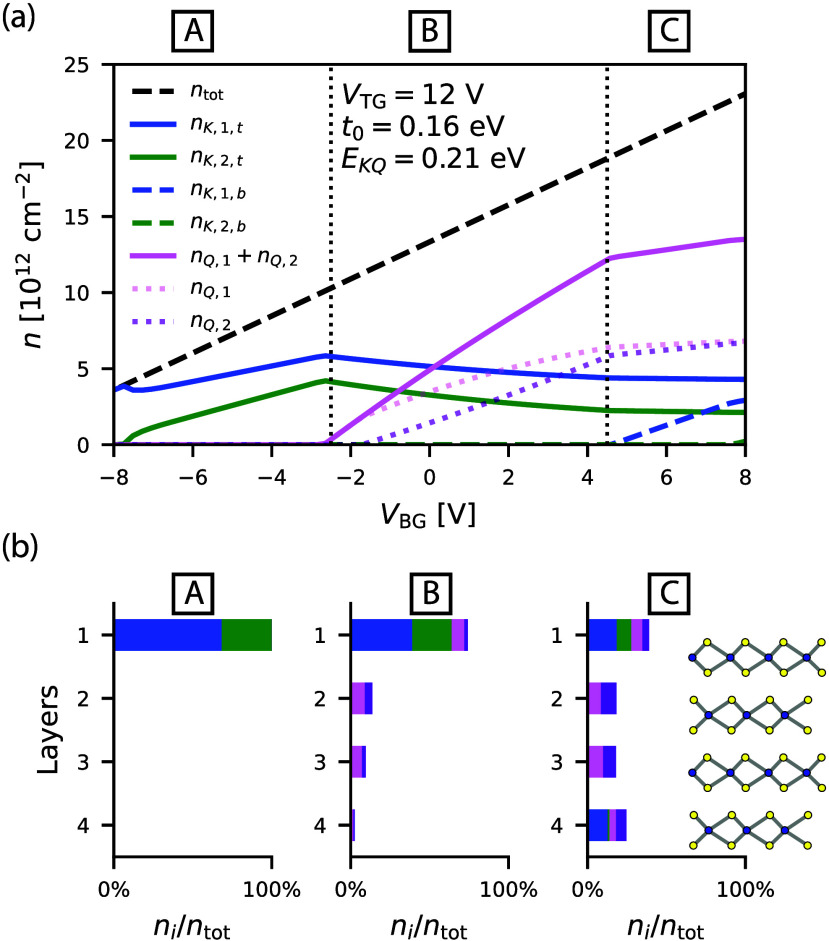
(a) Band densities obtained from the theoretical model using the parameters *E*_*QK*_ = 0.21 eV and *t*_0_ = 0.16 eV. The densities are plotted as a function of *V*_BG_ at *V*_TG_ = 12 V, enabling a direct comparison with Figure 2b. The densities in the *K* valleys of the top layer (layer 1), *n*_*K*,1,*t*_ and *n*_*K*,2,*t*_, are shown as solid blue and green lines, respectively. The *K* valley densities in the bottom layer (layer 4) are depicted with dashed lines following the same color code. The total density in the *Q* valleys (*n*_*Q*,1_ + *n*_*Q*,2_ for a direct comparison with the experiment) is represented by the pink solid line, while the individual components are shown by the dotted lines. (b) Percentages of the total density distributed across the four MoS_2_ layers with the different bands encoded by the colors (consistent with panel a). As a representation of the three regimes, we selected specific gate voltage configurations: (*V*_TG_ = 12 V, *V*_BG_ = −6 V) for regime A, (*V*_TG_ = 12 V, *V*_BG_ = 0 V) for regime B, and (*V*_TG_ = 12 V, *V*_BG_ = 8 V) for regime C.

This model successfully captures the gate voltage dependence of the valley-specific densities and provides insights into the layer distribution of electron densities. In regime B, the model predicts an asymmetric density distribution across the layers, which results in a spin degeneracy lifting of *Q* valley states when *D* ≠ 0, due to SOC (see the dotted lines in Figure 3a). This prediction aligns with a detailed analysis of the FFT spectrum (see Supplementary Note 5), where we observe a splitting of the SdHO frequency corresponding to the residual density.

The experimentally observed splitting of the *Q* valley is notably less pronounced than that predicted by our model. This discrepancy may arise from crystal field effects at the interface with the hBN dielectrics.^23^ In the Supporting Information (see Supplementary Note 12), we account for the influence of hBN by introducing an on-site potential energy difference, Δ_hBN_, between the outer layers (which interface directly with hBN) and the inner layers. Incorporating this offset reduces the level of hybridization between the *Q* valleys of the outer and inner layers. This adjustment results in a more symmetric layer distribution of the *Q* valley electrons, thereby mitigating inversion symmetry breaking induced by the displacement field and leading to a weaker spin–orbit splitting. Consequently, the model’s prediction of spin–orbit splitting becomes more consistent with the experimental observations.

Next, we examine the effect of the top gate voltage, focusing on the densities in the *Q* and *K* valleys of the top layer. To track the top gate dependence of these densities, we present the FFT of Δρ_*xx*_(*B* ^–1^) at *V*_BG_ = 3.5 V for various *V*_TG_ values in Figure 4a. In this regime, the finite *V*_BG_ allows us to observe oscillations originating from both *Q* and *K* valley states of the top layer (highlighted in pink and blue, respectively) without inducing charges in the *K* valleys of the bottom layer. Figure 4b shows that the *K* valley frequency of the top layer increases with *V*_TG_, while the *Q* valley frequency is nearly constant due to interlayer screening. These data suggest that the top gate voltage induces a transition of the band edge from the *Q* valleys at low gate voltages to the *K* valleys at higher voltages.

**Figure 4:**
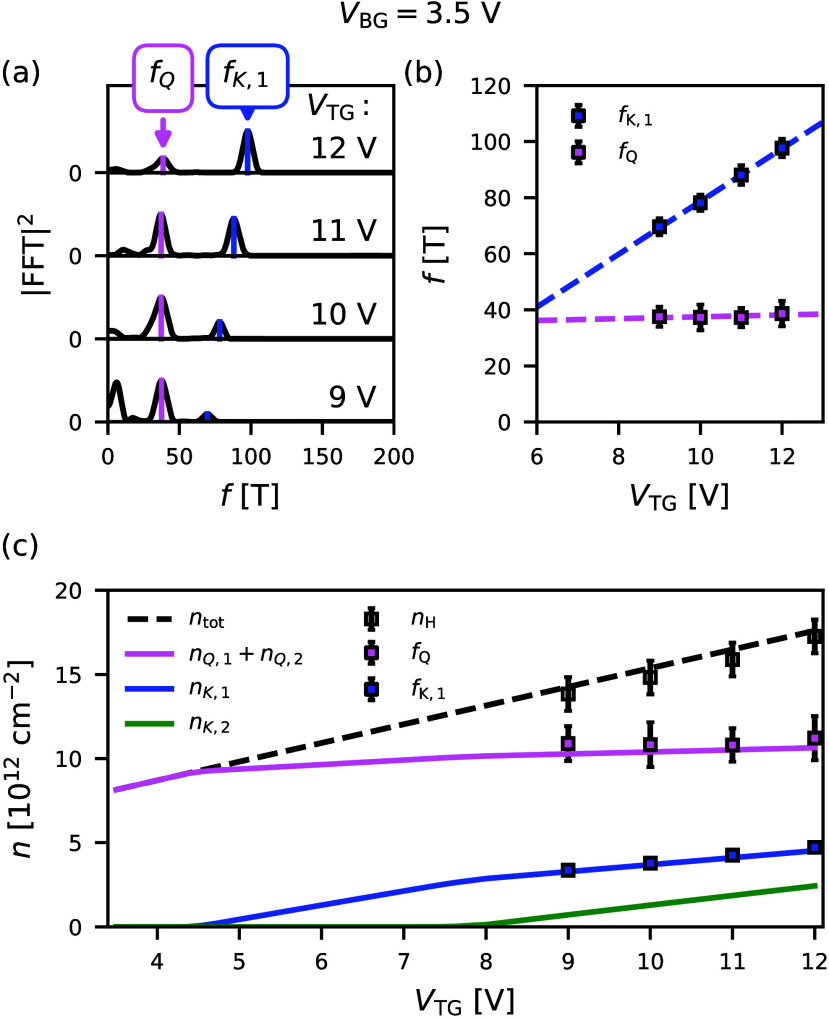
(a) FFT of Δρ_*xx*_(*B* ^–1^) at *V*_BG_ = 3.5 V and various *V*_TG_ values. The peaks associated with the *Q* and *K*_1_ valleys are highlighted by the vertical pink and blue lines, respectively. Note that the low-frequency component visible in the FFT arises from imperfect background subtraction and does not correspond to a physical valley population. (b) Frequencies obtained from panel a plotted as a function of *V*_TG_. (c) Comparison between the densities obtained from the model (solid lines) and the densities obtained from the experiment (square markers). The model parameters are the same as those of Figure 3.

In Figure 4c, we compare valley densities predicted by the model (solid lines) with experimental densities (square markers), showing good agreement. Due to high contact resistance, the low-*V*_TG_ regime is experimentally inaccessible, so we rely on model predictions for this range. According to the model, at low bias (*V*_TG_ < 4.5 V), the *Q* valley remains the only occupied band, confirming that the conduction band minimum in low-bias four-layer MoS_2_ is located at the *Q* point and demonstrating the presence of a gate-induced *Q*–*K* band edge transition.

Having established the relevance of the gate bias in determining the relative band alignment in multilayer MoS_2_, we extended our model to bilayer and trilayer systems (see Supplementary Note 13) to address discrepancies between experiments and DFT-calculated band structures. Using consistent model parameters, *E*_*KQ*_ = 0.21 eV and *t*_0_ = 0.16 eV, we qualitatively reproduce the experimental trends across different layer numbers.

For biased bilayer MoS_2_, the model predicts that the *K* valleys in top and bottom layers are occupied bands while the *Q* valleys are not, as observed in ref 10. In our earlier study on trilayer MoS_2_,^11^ we observed electron transport predominantly through the *K* valleys in the outer layers, although the band contributing to the electrons in the middle layer remained unidentified. Our model reveals the missing element in our previous work, showing that both *K* and *Q* valleys were populated, as observed in the four-layer case.

In conclusion, our study provides a comprehensive understanding of the gate-tunable band structure and valley occupation in four-layer MoS_2_ through a combination of magnetotransport experiments and a self-consistent hybrid *k*·*p* tight-binding model. At low gate voltages, we confirm that the conduction band minimum is centered at the *Q* point, which is in line with theoretical expectations for multilayer MoS_2_. However, as the gate voltage increases, charge redistribution toward the layer closest to the positive gate electrode induces a transition of the band edge from the *Q* valleys to the *K* valleys. By extending our model to bilayer and trilayer MoS_2_, we successfully bridge previous discrepancies between experimental observations^10,11^ and density functional theory predictions,^12–17^ underscoring the importance of interlayer screening and layer-specific charge accumulation in determining valley occupation. These findings highlight the intricate interplay between the *K* and *Q* valleys in multilayer MoS_2_, paving the way for future valleytronic and electronic applications that exploit layer-specific charge and valley control.

## Supplementary Material



## Notes

Details of device fabrication, additional magnetotransport characterization, Fourier analysis of Shubnikov–de Haas oscillations, comparison between different samples, and theoretical model description with parametric dependence analysis (PDF)
