# Supplementary material for: Gate-Tunable Band Edge in Few-Layer MoS2
Source: Nano Lett. 2025 Jun 22;25(26):10472–7. doi: 10.1021/acs.nanolett.5c01998 (PMC12232393; doi:10.1021/acs.nanolett.5c01998)
Supplement: Supplementary file 1 [file nl5c01998_si_001.pdf]

Gate-tunable band-edge in few-layer MoS_2

Supplementary information

Michele Masseroni[1], Isaac Soltero[2, 3], James G. McHugh[2, 3], Igor Rozhansky[2, 3], Xue Li[2, 3], Alexander Schmidhuber[1], Markus Niese[1], Takashi Taniguchi[4], Kenji Watanabe[5], Vladimir I. Fal'ko[2,3], Thomas Ihn[1], and Klaus Ensslin[1]

[1]Solid State Physics Laboratory, ETH Zürich, 8093 Zürich, Switzerland
[2]Department of Physics and Astronomy, University of Manchester, Oxford Road, Manchester, M13 9PL, United Kingdom
[3]National Graphene Institute, University of Manchester, Booth St. E., Manchester, M13 9PL, United Kingdom
[4]International Center for Materials Nanoarchitectonics, 1-1 Namiki, Tsukuba 305-0044, Japan
[5]Research Center for Functional Materials, 1-1 Namiki, Tsukuba 305-0044, Japan

May 23, 2025

Supplementary Note 1: Sample fabrication

The samples consist of gated Van der Waals heterostructures in which a four-layer MoS_2 serves as the electrically active layer. The MoS_2 flakes are obtained from a bulk crystal (SPI Supplies) via mechanical exfoliation inside a glove box with an argon atmosphere. To prevent contamination, the MoS_2 flakes are never exposed to ambient conditions.

The number of layers is identified through optical contrast measurements [1, 2, 3], and the thickness is verified using an atomic force microscope (AFM) after encapsulation in hexagonal boron nitride (hBN).

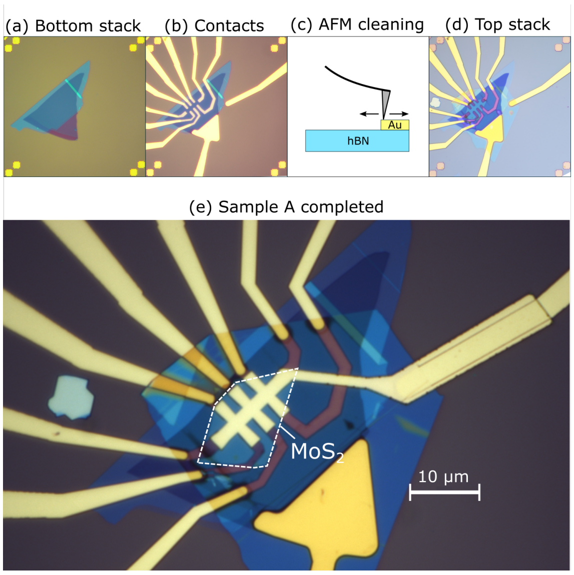

Figure 1: Overview of the fabrication process of Sample A. (a) The bottom stack, which consists of a hBN flake on a graphite flake is deposited on a Si/SiO_2 chip. (b) Metallic contacts (Ti/Au:3nm/12nm) are deposited on the bottom stack. (c) The tip of an AFM is used in contact mode to mechanically remove polymer residues from the fabrication process. (d) The top stack, which consists of a hBN and a four-layer MoS_2 flake, is aligned and deposited on the metallic contacts. (e) In the final step of the fabrication, a top gate is deposited on the VdW heterostructure.

The heterostructure is assembled in two steps using a polymer-based dry transfer technique [4, 5], following procedures similar to our previous studies [6, 7, 8]. An overview of the fabrication steps is shown in Fig.1.

First, we prepare the bottom part of the heterostructure (Fig. 1a), which is deposited on a silicon chip with a 285 nm layer of silicon oxide. The bottom part differs between the two samples: in sample A, it consists of hBN and a graphite flake, with the graphite serving as a bottom gate, whereas in sample B, the hBN is deposited on a pre-patterned metallic bottom gate. Next, the metal contacts (titanium/gold: 3 nm/12 nm) are fabricated using standard electron beam lithography , electron beam evaporation, and lift-off processes (Fig. 1b). Residual resist is mechanically removed using the tip of an AFM in contact mode (Fig. 1c). Subsequently, the second part of the heterostructure, consisting of the top hBN flake and the four-layer MoS_2, is aligned and deposited onto the pre-patterned contacts (Fig. 1d). Finally, a subsequent lithography process defines the metallic top gate in a Hall bar geometry (Fig. 1e).

Optical images of the samples are shown in the insets of Fig.9(a) and Fig.9(b) for samples A and B, respectively.

Supplementary Note 2: Gate transfer characteristics

This section discusses the gate transfer characteristics of the samples. These measurements provide insight into when the field-effect transistors turn on and off and offer an initial indication of the transparency of the metallic contacts.

In Fig. 2(a), we present the two-terminal conductance (G) of Sample A, measured at a temperature of $T = 1.3\,\mathrm{K}$. For this sample, finite conductance is achieved above the threshold voltage $V_{\mathrm{TG}}^0 \approx 7\,\mathrm{V}$. The large threshold voltage is necessary to accumulate a sufficient electron density in the MoS_2 layer, as electrons typically localize at densities below $1 \times 10^{12}\,\mathrm{cm}^{-2}$ [9]. Additionally, the electric displacement field modulates the Schottky barrier height at the metal-semiconductor interface. Both these effects suppress conductance at low gate voltages, making it challenging to disentangle their individual contributions.

In Fig.2(b), the conductance is shown as a function of V_{BG}, measured at $V_{\mathrm{TG}} = 11\,\mathrm{V}$. Applying a negative bottom gate voltage induces an insulating transition at $V_{\mathrm{BG}}^0 \approx -8.7\,\mathrm{V}$. The bottom gate voltage required to deplete the electron density does not directly depend on the threshold voltage V_{TG}^0, as V_{BG}^0 does not coincide with $(V_{\mathrm{TG}} - V_{\mathrm{TG}}^0) \times C_{\mathrm{T}}/C_{\mathrm{B}}$, where C_{T} and C_{B} are the top and bottom gate capacitances per unit area (see Tab.1). This observation suggests that a significant portion of V_{TG}^0 is required to overcome the Schottky barrier at the metal-MoS_2 interface, while doping in the MoS_2 is already induced by smaller gate voltages. Due to the presence of Schottky barriers, all experiments are conducted at finite top gate voltages, limiting the sample operation to nonzero displacement fields. Consequently, the displacement field is expected to influence the electron distribution among the different layers.

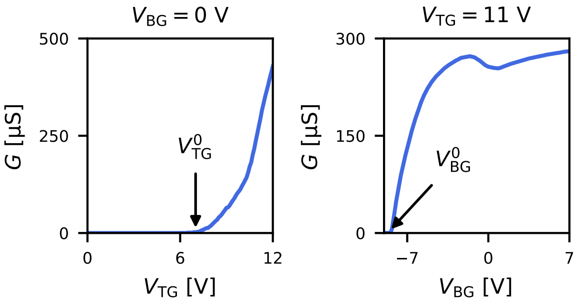

Figure 2: Gate transfer characteristics at $T = 1.3\,\mathrm{K}$ of sample A. (a) Two-terminal conductance as a function of V_{TG} at $V_{\mathrm{BG}} = 0$. The onset voltage is marked by $V_{\mathrm{TG}}^0 \approx 7\,\mathrm{V}$. (b) Two-terminal conductance as a function of V_{BG} at $V_{\mathrm{TG}} = 11\,\mathrm{V}$. The onset of conductance is marked by V_{BG}^0.

Table 1: Gate capacitance per area obtained by the slope of the Hall density as a function of the respective top and bottom gate voltages. The capacitance obtained for different gate voltage configurations shows slight deviations. The value reported in the table is the average of the capacitance obtained for different gate voltage configurations and the error is estimated from the standard deviation (σ).

Sample	C_{T} [nF cm^{-2}]	C_{B} [nF cm^{-2}]
A	178 ± 6	195 ± 2
B	146 ± 3	231 ± 7

Supplementary Note 3: Fast Fourier Transform of SdHO

In this section, we describe the numerical Fast Fourier Transformation (FFT) applied to analyze the Shubnikov–de Haas oscillations (SdHO) observed in the longitudinal magnetoresistance data presented in Fig.2(a) of the main text.

To extract the frequencies contributing to the SdHO, we perform a numerical FFT on the magnetoresistance data, processing each line of V_{BG} individually. As an example, we focus first on the trace in Fig. 3(a), which shows R_{xx} plotted against B at $V_{\mathrm{BG}} = -1.7\,\mathrm{V}$. The raw data exhibits a slow-varying negative background in the magnetoresistance. To remove this background, we fit and subtract a polynomial (typically 2nd or 4th order) from the raw data [1], and plot it against $1/B$ in Fig.3(b), as SdHO are periodic in $1/B$.

Since the FFT algorithm requires a uniform sampling rate in $1/B$, we interpolate the data and convert it to a constant sampling rate. A window function (Hamming function) is applied to the interpolated data to minimize spectral leakage, followed by zero-padding before performing the FFT to enhance frequency resolution.

The normalized FFT power spectrum, shown in Fig. 3(c), reveals two primary frequencies, $f_{K,1}$ and $f_{K,2}$, along with combination frequencies ($f_{K,1} + f_{K,2}$ and $f_{K,1} - f_{K,2}$) and higher-order harmonics. The presence of these two dominant frequencies is also apparent in the raw data (panel a), manifesting as a beating pattern in $R_{xx}(B)$, which results from the interference between these two frequencies.

The analysis is repeated for each V_{BG}, producing a two-dimensional Fourier spectrum, as shown in Fig. 4. Here, we plot the amplitude rather than the power spectrum to enhance contrast for the weaker peaks. In addition to the previously identified frequencies (also present for negative V_{BG}), we observe the emergence of a new frequency, f_Q, at positive V_{BG}. This frequency corresponds to the Landau fan frequency shown in Fig.2(c) of the main text.

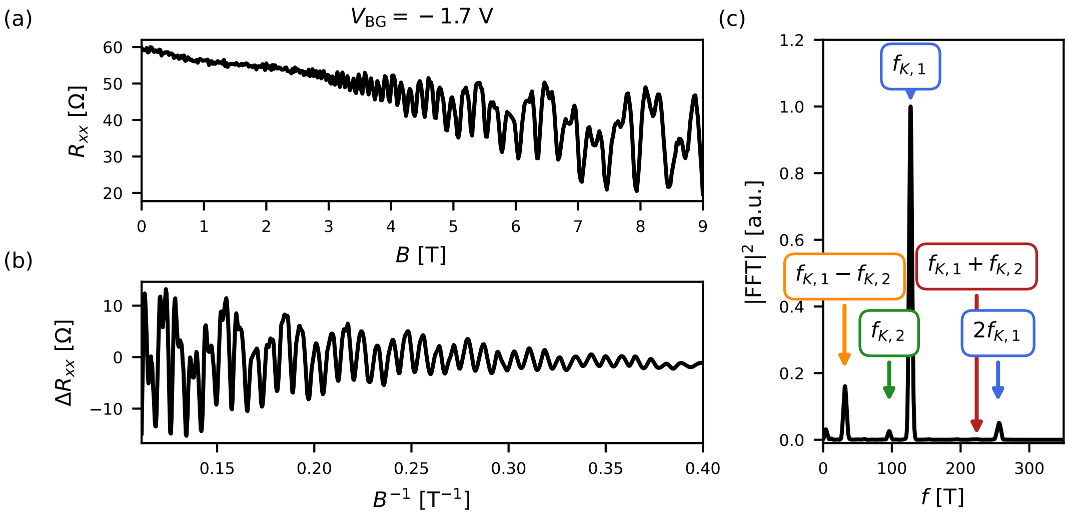

Figure 3: (a) Longitudinal resistance R_{xx} as a function of B at $V_{\mathrm{TG}} = 12\,\mathrm{V}$ and $V_{\mathrm{BG}} = -1.7\,\mathrm{V}$. (b) ΔR_{xx}, after subtracting a polynomial background, as a function of the inverse magnetic field. (c) FFT of the trace shown in (b). The labels identify the two main frequencies, $f_{K,1}$ and $f_{K,2}$, the sum and difference of these frequencies and their higher harmonics.

[1] An alternative background subtraction method involves computing the numerical derivative $\mathrm{d}R_{xx}/\mathrm{d}B$.

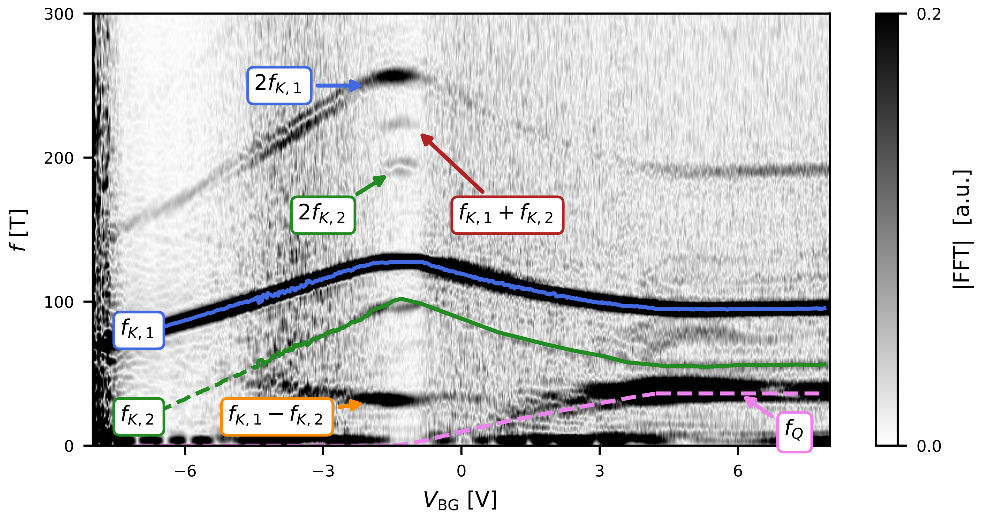

Figure 4: FFT of the magentoresistance at $V_{\mathrm{TG}} = 12\,\mathrm{V}$ shown in Fig.2(a) of the main text. The colorbar corresponds to the Amplitude of the Fourier spectrum normalized, line-by-line for each V_{BG}, to the maximum amplitude (corresponding in this case to the amplitude of the oscillations with frequency $f_{K,1}$). The labels identifies the main frequencies in the spectrum, $f_{K,1}$, $f_{K,2}$ and f_Q, as well as their combinations and higher harmonics. The solid lines are obtained from the peaks of the FFT, while the dashed lines are obtained from charge conservation arguments (see discussion in the text).

Supplementary Note 4: Localization density and non-linear Hall density

In this section, we analyze the insulating phase induced by applying a negative voltage to the bottom gate. In Fig.5, we replot the conductance data (blue curve) from Fig.2(b) on a logarithmic scale, alongside the electron density extracted from Hall effect measurements (red curve). Interestingly, the sharp drop in conductance at V_{BG}^0 does not align with the linear extrapolation of the Hall density to zero, indicating that electrons localize at a finite density.

From the conductance G asymptotically approaching zero near $V_{\mathrm{BG}} \approx -8.7\,\mathrm{V}$, we estimate the localization density to be approximately $n \approx 1.5 \times 10^{12}\,\mathrm{cm}^{-2}$. This localization density is attributed to intrinsic defects in the MoS_2 crystal, as similar values are consistently observed across our MoS_2 samples [9].

Localized states at the bottom of the conduction band provide valuable insights into band occupation. These states can be effectively considered zero-mobility states, which means that they do not contribute to the Hall effect. Consequently, the filling of these localized states leads to a saturation of the Hall density, as observed near $V_{\mathrm{BG}} = 0$ in Fig. 5.

After the localized states are fully saturated, delocalized states begin to fill, resulting in an increase in the Hall density and the recovery of the linear trend expected from a parallel-plate capacitor model. From the Hall density plateau near $V_{\mathrm{BG}} = 0$, we estimate a density of approximately $2 \times 10^{12}\,\mathrm{cm}^{-2}$, consistent with the localization density inferred from the conductance data.

This consistency between the conductance data and the Hall effect supports the conclusion that the localized states play a crucial role in determining the low-density transport behavior in MoS_2.

Note on the Hall Resistance non-Linearity and carrier density extraction

In dual-gated multilayer MoS_2, we observe that the Hall resistance (R_{xy}) is not strictly linear at low magnetic fields, particularly in regimes where multiple valleys (K and Q) are simultaneously occupied. To illustrate this behavior, Fig. 6 presents representative $R_{xy}(B)$ traces measured in the three regimes defined in the main text: A (K-valley only), B (K and Q valleys), and C (further band occupation).

In regime A, R_{xy} remains approximately linear despite the presence of spin-orbit split K-valleys, indicating comparable mobilities of the carriers. In contrast, regimes B and C show clear non-linearities at low fields, consistent with a multiband transport scenario involving valleys with distinct mobilities.

To accurately extract the total carrier density n_{tot} in such regimes, we utilize the asymptotic behavior of the Hall resistance. A two-band Drude model, which can be extended to more bands, shows that R_{xy} approaches $1/(en_{\mathrm{tot}})$ at high magnetic fields. Therefore, we determine n_{tot} by fitting the linear region of $R_{xy}(B)$ for $B > 1\,\mathrm{T}$,

where non-linear contributions become negligible.

This procedure ensures reliable density estimates across all regimes, despite the complexity introduced by multiband occupation.

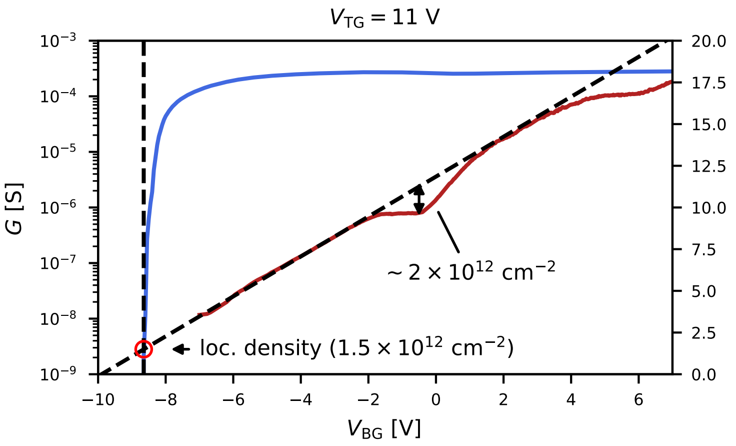

Figure 5: Comparison between conductance (left axis) and Hall density (right axis) at $V_{TG} = 11\,V$. The conductance exponentially converges to zero at $V_{BG} \approx -8.7\,V$, indicating the localization of electrons in the MoS_2 sample. The localization density is $n \approx 1.5 \times 10^{12}\,cm^{-2}$.

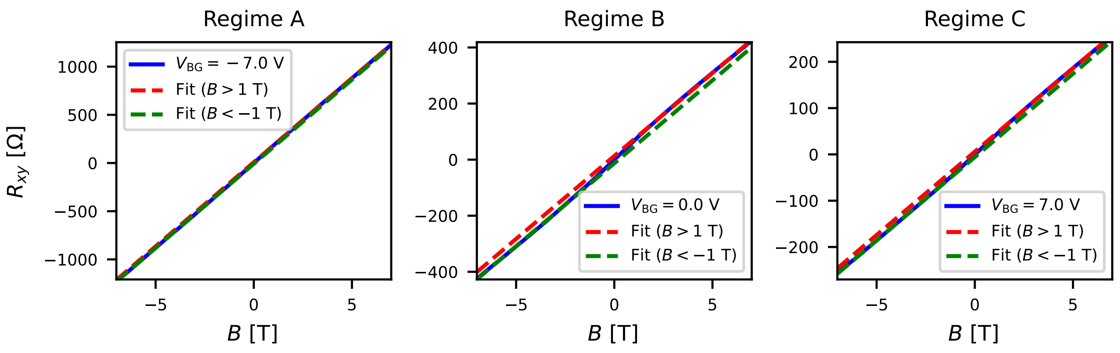

Figure 6: Hall resistance R_{xy} as a function of magnetic field B for representative gate configurations in regimes A, B, and C (as defined in the main text). In regime A, the response is approximately linear, whereas in regimes B and C, clear non-linearities appear at low fields due to the occupation of Q valleys and the presence of multiple carrier populations with differing mobilities. The high-field linear region (typically $B > 1\,T$) is used to extract the total carrier density.

Supplementary Note 5: Spin-orbit splitting of the Q valley

In the main text, we associate the frequency labeled f_Q in Fig.4 with the electron density in the Q valley, based on the large degeneracy ($g = 12$) inferred from the SdHO. A closer examination of the FFT spectrum in Fig.7 reveals a splitting of the peak associated with the Q valley frequency, indicating a lifting of the degeneracy.

In an unbiased MoS_2 sample with an even number of layers, inversion symmetry is preserved, resulting in a degeneracy of $g = 12$, which originates from a sixfold valley degeneracy and a twofold spin degeneracy. However, the applied external electric field breaks the inversion symmetry. As a result, the intrinsic spin-orbit coupling (SOC) manifests as a lifting of the spin degeneracy due to this symmetry breaking. Therefore, we attribute the observed peak splitting in the FFT spectrum to the spin-orbit splitting of the Q valley.

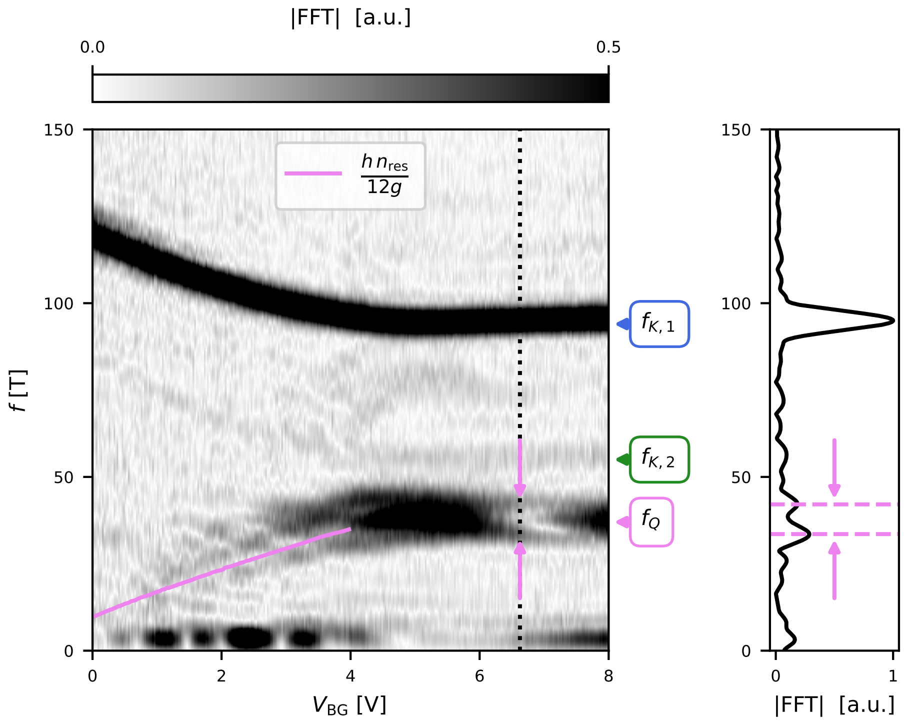

Figure 7: FFT of the data shown in Fig.2(c) of the main text. A closer look the peak associated with the density in the Q valleys reveals a splitting. The pink solid line is obtained by rescaling the residual density according to $h\,n_{\mathrm{res}}/(12e)$. The line fall between the split frequencies, as this represents the average of the two frequencies. The additional frequencies are related to the densities in the K valleys, as labeled in the figure. The right panel shows a linecut at constant V_{BG} (dotte vertical line in the left panel).

Supplementary Note 6: Top gate dependence and inter-layer screening

Our experiments reveal pronounced inter-layer screening effects, particularly evident in Fig. 2(c) of the main text, where the Landau fans for both the Q and K valleys saturate upon entering regime C ($V_{\mathrm{BG}} > 4\,\mathrm{V}$). As demonstrated by our theoretical model, this regime corresponds to the filling of the K valleys in the bottom layer, which introduces inter-layer screening. The onset of additional band filling at $V_{\mathrm{BG}} \approx 4\,\mathrm{V}$ is further corroborated by a dip in the Hall density, as shown in Fig. 2(b) of the main text.

Similar screening effects are observed as a function of the top gate voltage. In Fig.8(a), a line cut from Fig.2(c) of the main text at $V_{\mathrm{BG}} = 3.5\,\mathrm{V}$ is displayed. The corresponding FFT spectrum in Fig.8(b) reveals distinct frequencies attributed to the K and Q valleys. By analyzing the FFT data across a range of V_{TG} values, we track how the K and Q valley states respond to the top gate, as shown in Fig.8(c).

The results indicate that while the K valley frequency increases with V_{TG}, the Q valley frequency remains nearly unchanged. This behavior suggests that the Q valley states are effectively shielded from the electric field due to charge accumulation in the K valleys of the outer layers.

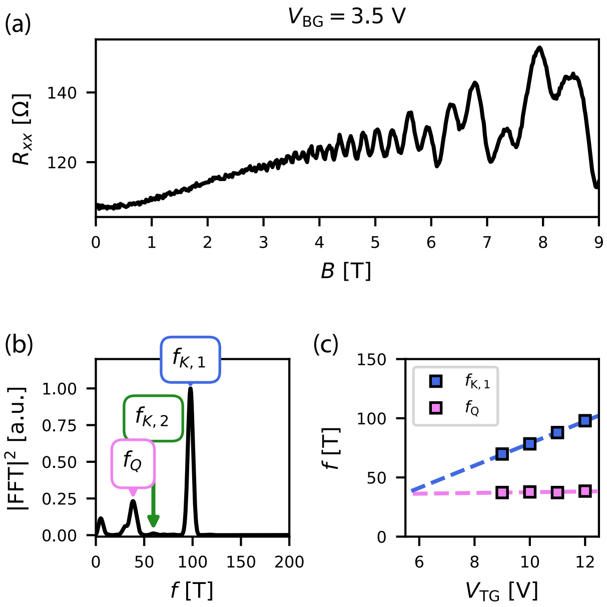

Figure 8: (a) R_{xx} plotted against B at $V_{\mathrm{TG}} = 12\,\mathrm{V}$ and $V_{\mathrm{BG}} = 3.5\,\mathrm{V}$. (b) FFT of $R_{xx}(B^{-1})$ after subtracting a polynomial background. The arrows labels highlight the frequencies $f_{K,i}$ (f_Q) stemming from the K (Q) valleys. (c) Frequencies extracted from the FFT for various V_{TG}. The dashed lines are linear fits to the data.

Supplementary Note 7: Sample comparison

We conducted experiments on two distinct samples: Sample A, which utilizes a graphite bottom gate, and Sample B, which features a metallic bottom gate. Optical images of these samples are presented in the insets of Fig. 9(a) and (b). This section provides a direct comparison of the main results obtained for Sample A (left columns) and Sample B (right columns).

Figures 9(a) and (b) show the longitudinal resistance, R_{xx}, as a function of V_{BG} at a fixed top gate voltage $V_{\mathrm{TG}} = 12\,\mathrm{V}$. While the gate capacitances differ between the two samples, the focus here is on qualitative rather than quantitative comparison. Both samples exhibit a non-monotonic dependence of R_{xx} on V_{BG}. Figures 9(c) and (d) display the electron density derived from the Hall resistance. For both samples, the Hall density reveals two distinct saturation points: one near $V_{\mathrm{BG}} \approx 0$, corresponding to the population of the Q valley (as discussed in the main text), and another at $V_{\mathrm{BG}} > 0$, which indicates the filling of the K valleys in the bottom layer. Sample A demonstrates overall superior electronic properties compared to Sample B. It achieves higher peak mobilities ($\mu_{\mathrm{A}} \approx 1.5 \times 10^4\,\mathrm{cm}^2(\mathrm{V,s})^{-1}$ versus $\mu_{\mathrm{B}} \approx 5.8 \times 10^3\,\mathrm{cm}^2(\mathrm{V,s})^{-1}$), reflecting its enhanced transport performance.

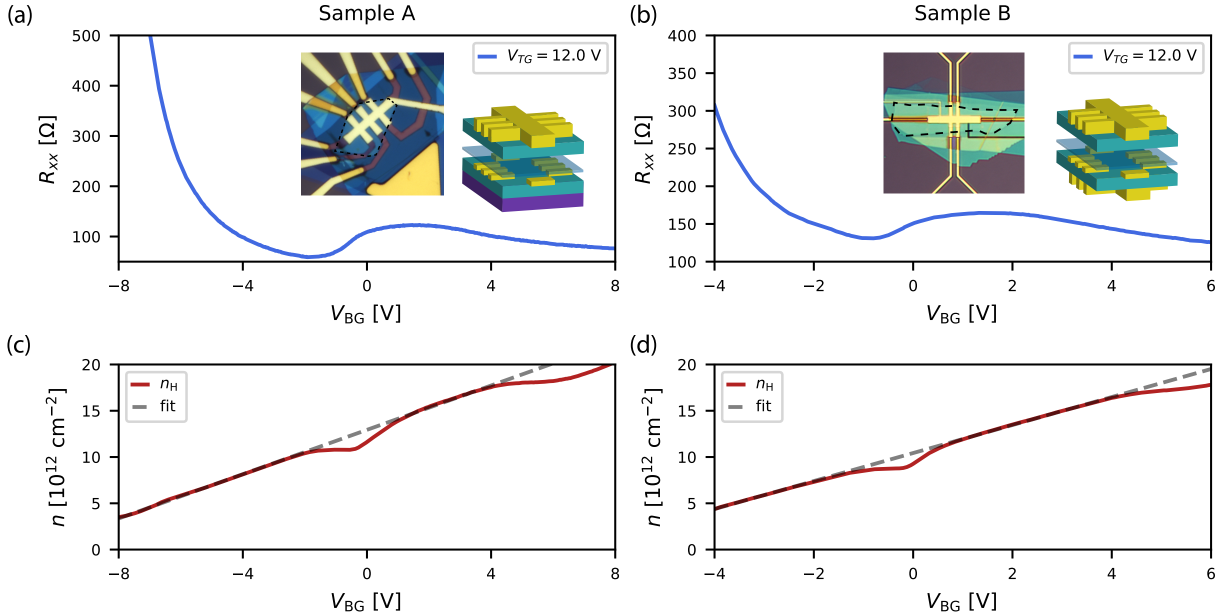

Figure 9: Comparison between Sample A (left column) and Sample B (right column). (a-b) R_{xx} measured as a function of V_{BG} at a constant $V_{\mathrm{TG}} = 12\,\mathrm{V}$. The insets show an optical image and a schematic view of the samples. (c-d) Hall density as a function of V_{BG} at $V_{\mathrm{TG}} = 12\,\mathrm{V}$.

In Fig. 10(a) and (b), we present the magnetoresistance data for Sample A and Sample B, respectively. The higher mobility of Sample A is evident in the larger amplitudes of the SdHO at comparable magnetic fields, as well as the onset of oscillations at lower B values. Despite the differences in mobility, both samples exhibit clear evidence of two distinct Landau fans. This observation is further confirmed by the FFT analysis shown in Fig. 10(c) and (d), which highlights the frequencies corresponding to these oscillations.

In Fig. 11(a) and (b), we present the derivative $\mathrm{d}R_{xx}/\mathrm{d}V_{\mathrm{BG}}$ as a function of the top and bottom gate voltages for Samples A and B, respectively. These measurements were taken under finite magnetic fields to examine how the SdHO depend on both gates. The minima in R_{xx} correspond to lines of constant band densities, and the derivative enhances the contrast of oscillations originating from the Q valley. In region A, we observe lines with a negative slope, corresponding to SdHO in the K valleys. The slope is determined by the ratio of the top and bottom gate capacitances, as both gates affect the same electron density. When V_{BG} enters regime B, the slope of the SdHO in the K valleys reverses sign. This reversal is attributed to a reduction in the displacement field across the four-layer MoS_2, which lowers the energy offset between the K and Q valleys. Once the K and Q valleys align energetically, charge begins to transfer into the Q valleys, reducing the density in the K valleys and thus causing the negative slope. In regime B, the oscillations corresponding to the Q valleys appear as vertical lines in the $(V_{\mathrm{BG}}, V_{\mathrm{TG}})$ plane, indicating that the density in the Q valleys is independent of V_{TG}. This behavior is attributed to inter-layer screening by the electron density in the K valleys of the top layer. Finally, in regime C, the K valleys in the bottom layer begin to fill, as confirmed by our theoretical model. The electron density in this newly occupied band screens the bottom gate, leading to a saturation of charge densities in the other bands.

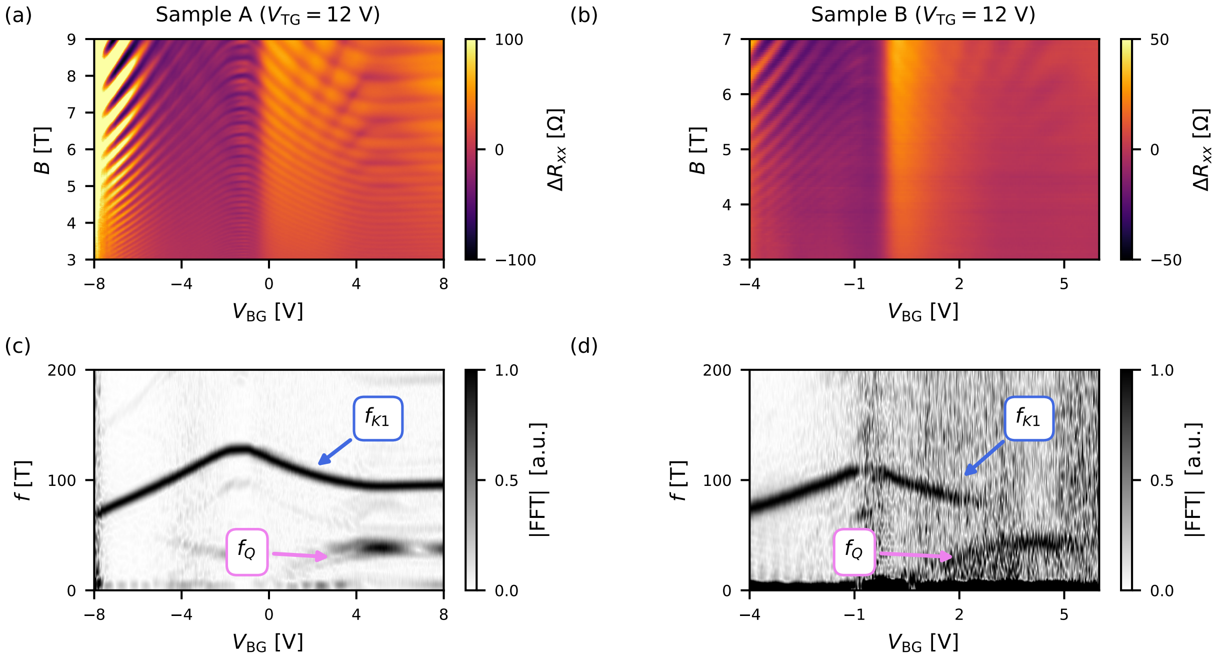

Figure 10: Comparison between Sample A (left column) and Sample B (right column). (a-b) ΔR_{xx} plotted against V_{BG} and B at $V_{\mathrm{TG}} = 12\,\mathrm{V}$. (c-d) FFT of $\Delta R_{xx}(B^{-1})$. The arrows highlight the SdHO frequency of the K and Q valleys.

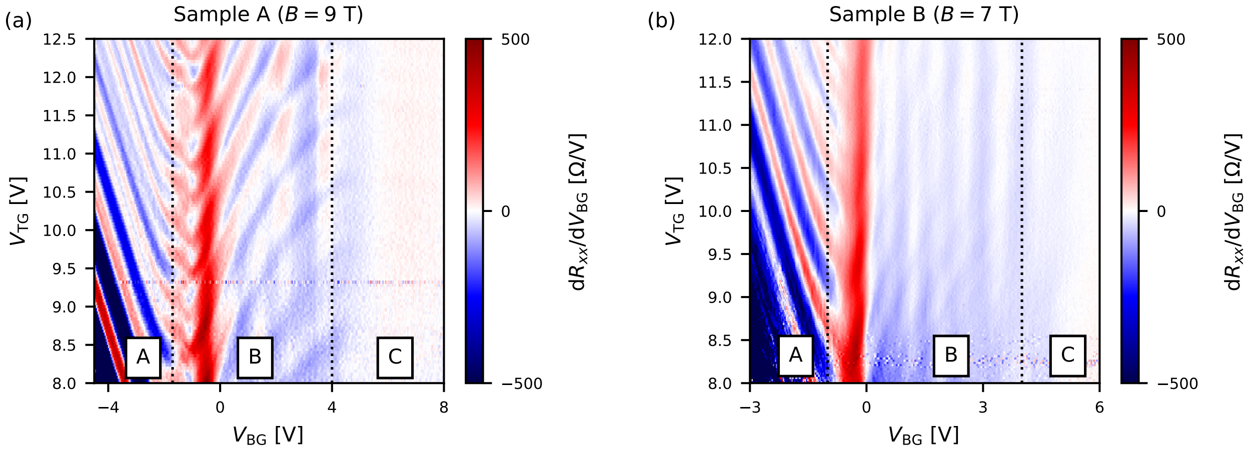

Figure 11: (a) Top gate vs bottom gate map of the transresistance of Sample A. The data were collected at a constant magnetic field of $B = 9\,\mathrm{T}$ and temperature $T \approx 100\,\mathrm{mK}$. The data are divided into three regime, as for the main text. (b) The same measurement as in panel (a), but for sample B. In this case the data were collected at $B = 7\,\mathrm{T}$ and $T = 1.3\,\mathrm{K}$.

Supplementary Note 8: Hybrid k·p tight-binding model for the conduction band near the Q point

To capture the interplay of spin-orbit coupling and interlayer hybridization of multilayer MoS_2 band edges, we employ a hybrid k·p tight-binding model. This combines k·p Hamiltonians of monolayer band edges, supplemented by minimal interlayer hopping terms, which we initially fit to first-principles density functional theory (DFT) calculations. Dispersion of band edge electrons is quantified as

$$E(k) = \frac{\hbar^2 k_x^2}{2m_{i,x}} + \frac{\hbar^2 k_y^2}{2m_{i,y}}, \tag{1}$$

where k is momentum relative to band edge minimum, and $m_{i,x/y}$ are the k_x, k_y-direction effective masses of electrons at band edge $i = K/Q$.

On top of this, spin-orbit coupling and interlayer hybridization are taken into account by the following Hamiltonian for an N-layer system:

$$\mathcal{H}_N = E_0^i \mathbb{I}_{2N} + \frac{\tau \Delta_{SO}^i}{2} \sum_{n=1}^{N} (-1^n) \sigma_z^n + t_0^i \sum_{n=1}^{N-1} \eta_x^n + H.c., \tag{2}$$

where E_0^i is the energy offset of band edge i, σ_z^n are Pauli matrices operating on spin on layer n, with -1^n accounting for alternating spin-orbit splitting of band edges in adjacent layers due to 2H stacking, τ is a valley index which accounts for opposite spin-splitting of Kramers pairs, and η_x^n are Pauli matrices operating in layer-space, generating off-diagonal hybridization between layers $n, n+1$. For example, for bilayer Q-point edges this gives the following 4×4 matrix:

$$\mathcal{H}_{BL} = \begin{pmatrix} E_0 + \Delta_{SO}^Q/2 & 0 & t_0 & 0 \\ 0 & E_0 - \Delta_{SO}^Q/2 & 0 & t_0 \\ t_0^* & 0 & E_0 - \Delta_{SO}^Q/2 & 0 \\ 0 & t_0^* & 0 & E_0 + \Delta_{SO}^Q/2 \end{pmatrix}, \tag{3}$$

operating in the basis $|\uparrow 1\rangle$, $|\downarrow 1\rangle$, $|\uparrow 2\rangle$, $|\downarrow 2\rangle$.

Supplementary Note 9: DFT parametrization

N	E_{KQ} [meV]	t_0^Q [meV]	Δ_{SO}^Q [meV]	Δ_{SO}^K [meV]
1	138.85	-	68.10	2.80
2	120.13	198.88	68.10	2.80
3	119.55	202.89	68.10	2.80
4	116.36	200.76	68.10	2.80

Table 2: Fits for energy parameters and spin-orbit couplings, at DFT-predicted equilibrium lattice parameters, $t_0^K \lesssim 1$ meV for all multilayers.

Hybrid model parameters were fitted to DFT calculations, which naturally incorporate spin–orbit coupling (SOC) and interlayer hybridization. Calculations including an applied out-of-plane displacement field across MoS_2 multilayers were also implemented to explicitly verify its on band edges. All calculations were performed using Quantum ESPRESSO with a plane-wave kinetic energy cutoff of 80 Ry for wavefunctions and 800 Ry for the charge density. A uniform Monkhorst-Pack k-point grid of $31 \times 31 \times 1$ sampling of the Brillouin zone was employed. The generalized gradient approximation (GGA) was employed for exchange-correlation effects, specifically using the Perdew-Burke-Ernzerhof (PBE) parametrization. Non-collinear SOC was included in all self-consistent field calculations with fully relativistic ultrasoft pseudopotentials. Van der Waals interactions were accounted for using the vdW-DF2-c09 correction, which effectively captures dispersion forces in layered materials, and produces particularly accurate in-plane and out-of-plane lattice constants, and ultrasoft pseudopotentials were used to approximate the interactions between nuclei and electrons.

We first extracted model parameters from MoS_2 multi-layers of different sizes, using structurally relaxed DFT atomic coordinates. We note that, as we take the K-point band edge energy as the zero in all parameterisations, the total set of parameters to fit are E_{KQ}, which is the relative offset of K and Q-point band edges, in addition to hybridization (t_0^i) and spin-splitting (Δ_{SO}^i) at each band edge. Fitted parameters from $N = 1-4$ layer MoS_2 band structures are listed in Table 2; we note a slight decrease in the K-Q offset in mutilayers due to the effect of remote bands, and highly consistent values for both offset and Q-point hybridization energies with increasing

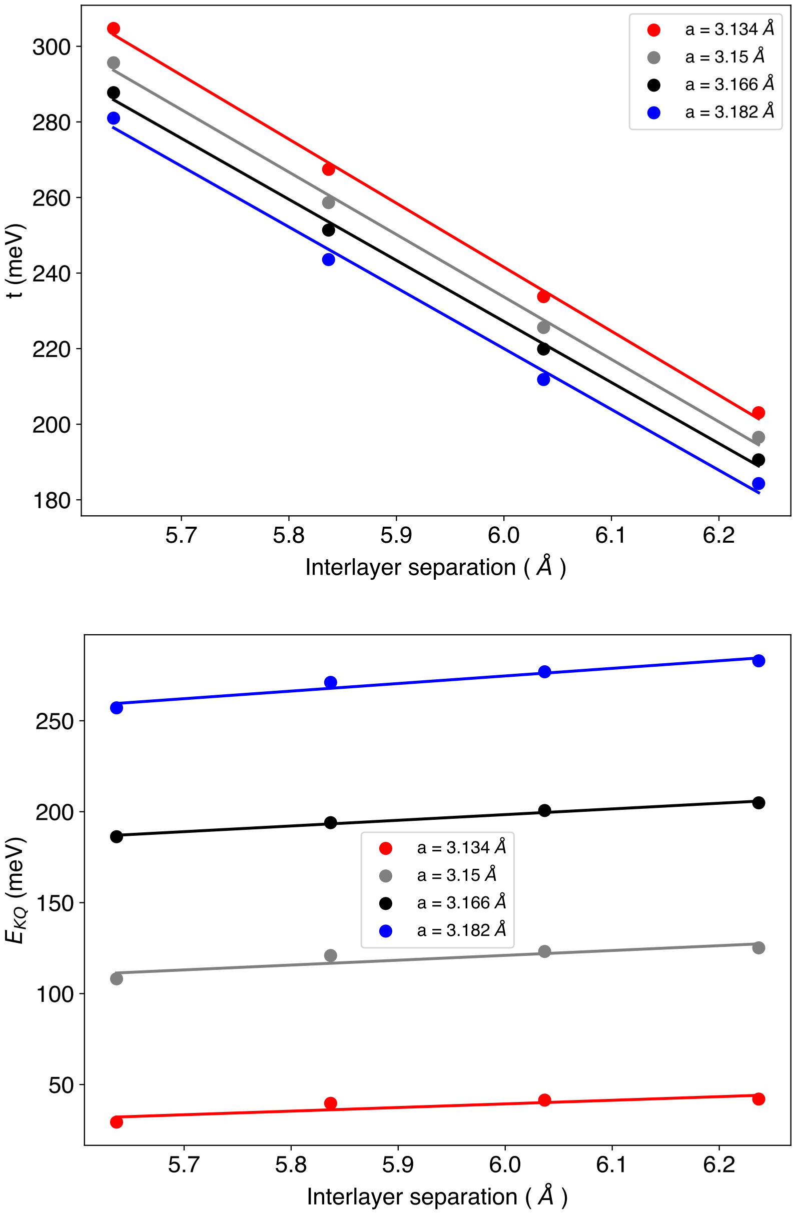

Figure 12: Variation of t_0^Q, E_{KQ} with in-plane and out-of-plane structural parameters.

numbers of layers for $N \geq 2$, as well as uniformly weak hybridization between K-point band edges. For this reason, we simply refer to t_0^Q as t_0 in the remaining sections.

Notably, the parameters determined in Table 2 are subject to the particular choice of DFT functional, and there is substantial variation in both in-plane lattice constant and interlayer separation between adjacent MoS_2 layers, which in turn has a substantial impact on both hybridization and offset. Due to this high degree of uncertainty, we further consider a range of values for in-plane strain and interlayer spacing of MoS_2-bilayers, to determine the plausible range for model parameter variation. Figure 12 shows calculated values of t_0^Q and E_{KQ}, which suggest values in the range $E_{KQ} = 100 - 200$ meV and $t_0^Q \lesssim 240$ meV.

Finally, to validate the influence of displacement field on the on-layer potential, we have also explicitly incorporated it in into DFT calculations of 4-layer MoS_2. This produced the expected dependence at both K- and Q-points through the incorporation of appropriate on-site energy shifts, $\propto edD/2\varepsilon_{zz}^{\mathrm{MoS_2}}$, with a consistent value of out-of-plane dielectric constant value $\varepsilon_{zz}^{\mathrm{MoS_2}} = 6.1$ in both K- and Q-edges.

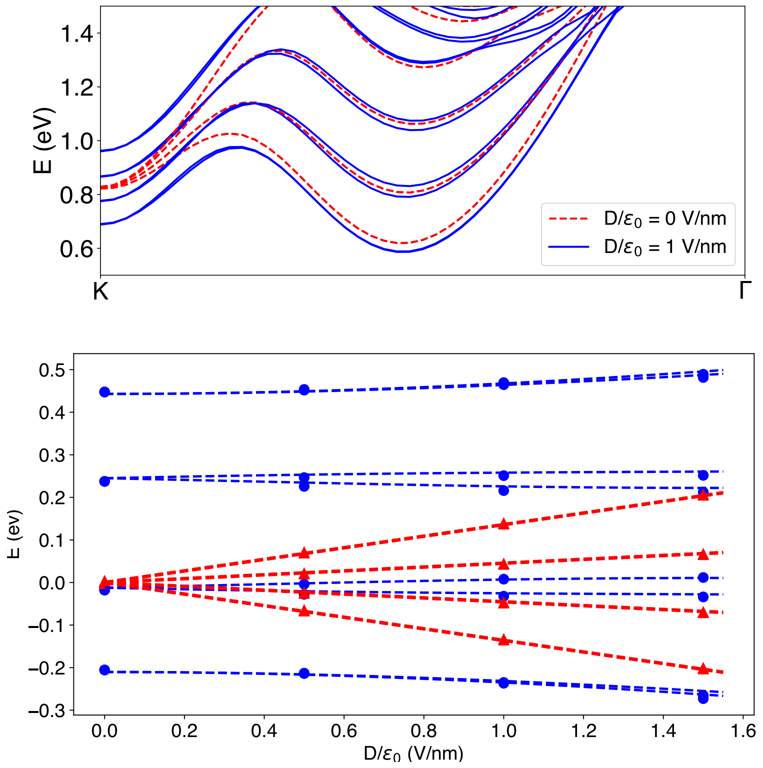

Figure 13: Top: DFT band structure, showing splitting at K- and Q-points in displacement fields exceeding $D/\epsilon_0 = 1$ V/nm. Bottom: DFT calculated eigenvalues at K (red) and Q (blue) point band edges as a function of displacement field, and corresponding model fits including an on-site potential term, with $\varepsilon_{zz}^{\mathrm{MoS_2}} = 6.1$.

Supplementary Note 10: Self-consistent screening analysis

In order to include the effects of internal electric fields introduced by charge redistribution across an N-layer system subject to a displacement field $D = (C_\mathrm{T}V_\mathrm{TG} - C_\mathrm{B}V_\mathrm{BG})/2\varepsilon_0$, we consider the on-layer potentials U_j $(1 \le j \le N)$ [10]:

$$U_j = U_1 + ed\left[\frac{(j-1)D}{\varepsilon_{zz}^{\mathrm{MoS}_2}} + \sum_{\ell=2}^{j} \mathcal{E}_{\ell-1,\ell}\right], \qquad j > 1, \tag{4a}$$

$$\mathcal{E}_{\ell-1,\ell} = \frac{e}{2\varepsilon_{zz}^{\mathrm{MoS}_2}\varepsilon_0}\left[\sum_{k\ge\ell} n_k - \sum_{k\le\ell-1} n_k\right], \tag{4b}$$

$$n_\mathrm{tot} = \frac{C_\mathrm{T}V_\mathrm{TG} + C_\mathrm{B}V_\mathrm{BG}}{e} = \sum_{k=1}^{N} n_k. \tag{4c}$$

Here, n_k is the total electron (number) density in layer k, $d = 0.612$ nm is the interlayer distance, and $\varepsilon_{zz}^{\mathrm{MoS}_2} = 6.1$ is the out-of-plane dielectric constant of bulk MoS_2 [11]. We fix the reference on-site energy in the first layer as $U_1 = -(N-1)edD/2\varepsilon_{zz}^{\mathrm{MoS}_2}$ in order to have a symmetrical effect of the displacement field over the system. The effective masses considered for Q valley electrons were fitted from the DFT calculations presented in Section , $m_{Q,x} = 0.51m_0$ and $m_{Q,y} = 0.75m_0$, while the spin-orbit splitting was taken from Table 2. The K valley effective masses for the lower and upper bands correspond to the values extracted from experimental data for MoS_2 monolayer, $m_{K,1} = 0.63m_0$ and $m_{K,2} = 0.84m_0$. According to the findings in Ref. [12], the spin-orbit splitting between K bands and the monolayer KQ band alignment undergoes significant renormalization due to electron-electron exchange interactions. In line with this framework, we introduce the layer-density functions $\Delta_{SO}^{K}(n_k)$ and $E_{KQ}(n_k)$ (see Fig. 14). The unrenormalized value of the K spin-orbit splitting considered is $\Delta_{\mathrm{SO},0}^{K} = 8$ meV, whereas E_{KQ} is taken as a parameter to be fixed.

Due to the absence of hybridization of the K valleys between layers, the on-site potentials U_j act as simple energy shifts, whereas for the Q valleys these potentials have to be incorporated in the diagonal terms of Eq. (2). Diagonalization of this Hamiltonian determines the band alignment between Q and K valleys, as well as the distribution of the total charge density n_tot across the layers. Since the layer densities n_k depend implicitly on the on-site potentials, these have to be calculated in a self-consistent fashion. As a first step, the total density is distributed equally over the all the layers, and in each iteration i we compute a new set of layer densities $\tilde{n}_k^{(i)}$, which are used to define the value of the densities for the next iteration as

$$n_k^{(i)} = \beta\tilde{n}_k^{(i)} + (1-\beta)n_k^{(i-1)}, \tag{5}$$

where $\beta = 5\times10^{-2}$ establishes the fraction of the density that is updated. In Fig. 15 we show the convergence of the layer densities and on-layer potentials as a function of number of iterations with gate configuration $V_\mathrm{TG} = 12$ V, $V_\mathrm{BG} = 3$ V and parameters $E_{KQ} = 0.18$ eV and $t_0 = 0.14$ eV.

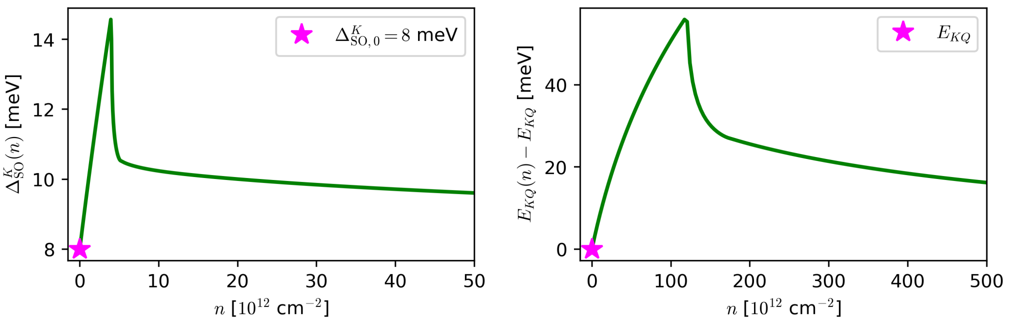

Figure 14: Density-dependent renormalization of spin-orbit splitting of K bands (left) and energy offset between K and Q bands (right) in monolayer MoS_2. The unrenormalized values are represented by stars.

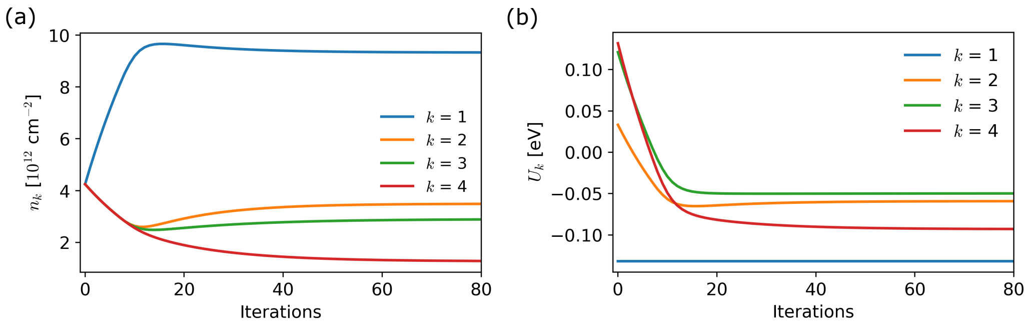

Figure 15: Convergence analysis for the layer densities n_k and on-layer potentials U_k with $V_{TG} = 12$ V and $V_{BG} = 3$ V, which corresponds to $n_{tot} = 17 \times 10^{12}$ cm^{-2} and $D = 0.88$ V/nm.

Supplementary Note 11: Analysis of the parametric dependence

The valley densities in each layer depend strongly on the choice of parameters E_{KQ} and t_0. We observe that, in order to obtain charge accumulation in the K valley of the first layer at high gate bias, either E_{KQ} has to be increased or t_0 decreased from the DFT predicted values. Furthermore, in Fig. 16, we see that for a fixed value of V_{TG}, the range of values for V_{BG} where we find a finite density contribution in the bottom layer is very sensitive to the relative values of the parameters. We fix these values to match the total Q valley density, $n_{Q,1} + n_{Q,2}$, at the crossover from regime B to regime C (described in the main text).

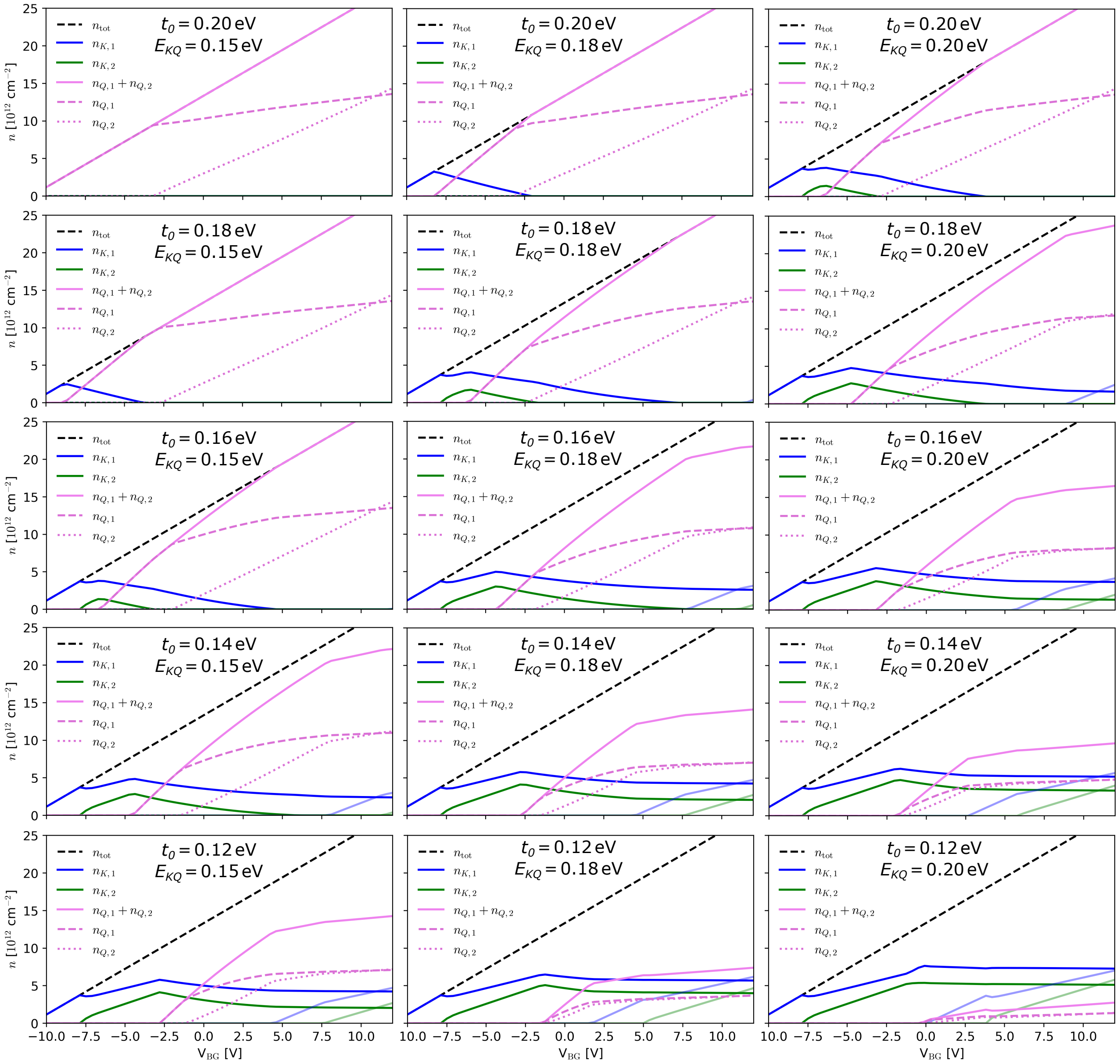

Figure 16: Band densities calculated from the self-consistent analysis for multiple values of the parameters E_{KQ} and t_0 as a function of V_{BG} for $V_{\mathrm{TG}} = 12$ V. Densities in the K valleys of the top layer, $n_{K,1}$ and $n_{K,2}$, are shown by solid blue and green lines, respectively, while the semi-transparent lines correspond to densities in the bottom layer. The density in the Q valleys $(n_{Q,1}, n_{Q,2})$ are represented by pink lines.

Supplementary Note 12: Effects of hBN encapsulation

The effect of the hBN environment is incorporated through an additional on-site potential energy shift Δ_{hBN} for the outer layers of the system in the Q valleys Hamiltonian. This energy shift is considered to be negligible on the K bands since the composition of these states is almost entirely d-orbitals from the metal atoms, in contrast with the Q bands, which have a finite contribution from the chalcogen p-orbitals.

In Fig. 17(a), we show the evolution of the valley populations as a function of V_{BG} with $V_{\mathrm{TG}} = 12$ V for three different values of Δ_{hBN}. This additional on-layer potential contribution leads to the redistribution of the Q band states towards the middle layers, producing nearly degenerate Q_1 and Q_2 bands, as observed in the experimental results (see Fig. 7). A similar scenario is observed in the analysis of the valley densities as a function of V_{TG} for $V_{\mathrm{BG}} = 3.5$ V, where the impact of the increasing bias gets suppressed for larger values of Δ_{hBN} due to the symmetrical distribution of Q_1 and Q_2 states over the four layers.

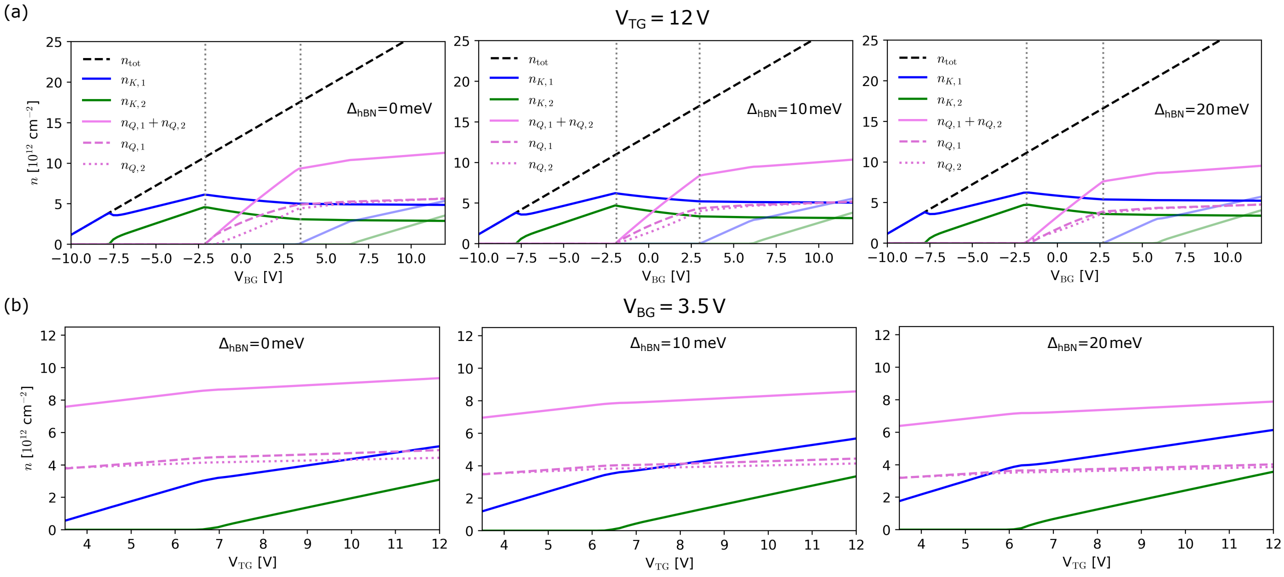

Figure 17: Band densities for multiple values of the on-site potentials Δ_{hBN} as a function of (a) V_{BG} with $V_{\mathrm{TG}} = 12$ V and (b) V_{TG} with $V_{\mathrm{BG}} = 3.5$ V. The parameters considered in the calculation are $E_{KQ} = 0.21$ eV and $t_0 = 0.16$ eV.

Supplementary Note 13: Application of the model to two- and three-layer systems

As an additional test to the validity of our choice of parameters E_{KQ} and t_0, we extend our analysis to two- and three-layer MoS_2 by implementing the self-consistent screening analysis (Eqs. (4)) together with the Q valley Hamiltonians for $N = 2$ and $N = 3$. The gate dependent valley densities in bilayer MoS_2 show that, for large enough gate bias, the charge density is accumulated completely in the K valleys of one of the layers. As the bias is continuously decreased, the density in this layer reaches saturation and K valleys of the second layer begin to populate. Furthermore, we obtained a null density of the Q valleys for the range of total densities and gate bias analyzed. These results are in full agreement with the experimental results reported in Ref. [7].

The qualitative picture obtained for three-layer MoS_2 has great similarity with the results obtained for four layers, where we can identify the same three regimes for the interplay between K and Q densities as a result from the asymmetrical on-layer potential distribution. This complements the analysis presented in Ref. [8].

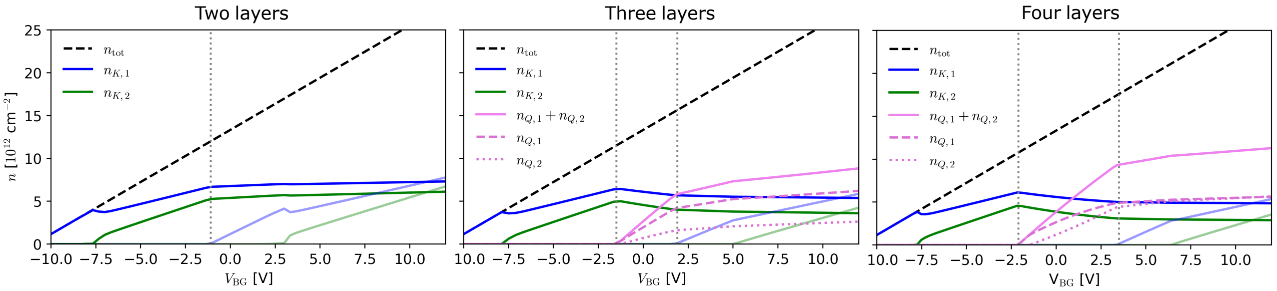

Figure 18: Band densities for two-, three- and four-layers systems as a function of $V_{\rm BG}$ with $V_{\rm TG} = 12$ V. The parameters considered in the calculation are $E_{KQ} = 0.21$ eV, $t_0 = 0.16$ eV and $\Delta_{\rm hBN} = 0$ meV.

References

[1] Huanping Yang, Hailong Hu, Yingying Wang, and Ting Yu. Rapid and non-destructive identification of graphene oxide thickness using white light contrast spectroscopy. *Carbon*, 52:528–534, 2013.

[2] Dan Bing, Yingying Wang, Jing Bai, Ruxia Du, Guoqing Wu, and Liyan Liu. Optical contrast for identifying the thickness of two-dimensional materials. *Optics Communications*, 406:128–138, January 2018.

[3] Mainak Mondal, Ajit K. Dash, and Akshay Singh. Optical microscope based universal parameter for identifying layer number in two-dimensional materials. *ACS Nano*, 16(9):14456–14462, 2022.

[4] Andres Castellanos-Gomez, Michele Buscema, Rianda Molenaar, Vibhor Singh, Laurens Janssen, Herre S. J. van der Zant, and Gary A. Steele. Deterministic transfer of two-dimensional materials by all-dry viscoelastic stamping. *2D Materials*, 1(1):011002, 2014.

[5] Filippo Pizzocchero, Lene Gammelgaard, Bjarke S. Jessen, José M. Caridad, Lei Wang, James Hone, Peter Bøggild, and Timothy J. Booth. The hot pick-up technique for batch assembly of van der waals heterostructures. *Nature Communications*, 7(1):11894, 2016.

[6] Riccardo Pisoni, Andor Kormányos, Matthew Brooks, Zijin Lei, Patrick Back, Marius Eich, Hiske Overweg, Yongjin Lee, Peter Rickhaus, Kenji Watanabe, Takashi Taniguchi, Atac Imamoglu, Guido Burkard, Thomas Ihn, and Klaus Ensslin. Interactions and magnetotransport through spin-valley coupled landau levels in monolayer MoS_2. *Physical Review Letters*, 121(24):247701, 2018.

[7] Riccardo Pisoni, Tim Davatz, Kenji Watanabe, Takashi Taniguchi, Thomas Ihn, and Klaus Ensslin. Absence of Interlayer Tunnel Coupling of K-Valley Electrons in Bilayer MoS_2. *Physical Review Letters*, 123(11):117702, September 2019.

[8] Michele Masseroni, Tim Davatz, Riccardo Pisoni, Folkert K. de Vries, Peter Rickhaus, Takashi Taniguchi, Kenji Watanabe, Vladimir Fal'ko, Thomas Ihn, and Klaus Ensslin. Electron transport in dual-gated three-layer MoS_2. *Physical Review Research*, 3(2):023047, April 2021.

[9] Michele Masseroni, Tingyu Qu, Takashi Taniguchi, Kenji Watanabe, Thomas Ihn, and Klaus Ensslin. Evidence of the coulomb gap in the density of states of mos_2. *Phys. Rev. Res.*, 5:013113, Feb 2023.

[10] David A Ruiz-Tijerina, Mark Danovich, Celal Yelgel, Viktor Zólyomi, and Vladimir I Fal'ko. Hybrid k·p tight-binding model for subbands and infrared intersubband optics in few-layer films of transition-metal dichalcogenides: MoS_2, $MoSe_2$, WS_2, and WSe_2. *Physical Review B*, 98(3):035411, 2018.

[11] Fábio Ferreira, VV Enaldiev, and VI Fal'ko. Scaleability of dielectric susceptibility ε_{zz} with the number of layers and additivity of ferroelectric polarization in van der Waals semiconductors. *Physical Review B*, 106(12):125408, 2022.

[12] Igor Rozhansky and Vladimir Fal'ko. Exchange-enhanced spin-orbit splitting and its density dependence for electrons in monolayer transition metal dichalcogenides. *Phys. Rev. B*, 110:L161404, Oct 2024.
